# Mechanically robust stretchable organic optoelectronic devices built using a simple and universal stencil-pattern transferring technology

**DOI:** 10.1038/s41377-018-0041-x

**Published:** 2018-07-04

**Authors:** Da Yin, Nai-Rong Jiang, Yue-Feng Liu, Xu-Lin Zhang, Ai-Wu Li, Jing Feng, Hong-Bo Sun

**Affiliations:** 1 State Key Laboratory of Integrated Optoelectronics, College of Electronic Science and Engineering, Jilin University, 2699 Qianjin Street, 130012 Changchun, China; 2 State Key Laboratory of Precision Measurement Technology and Instruments, Department of Precision Instrument, Tsinghua University, Haidian 100084 Beijing, China

## Abstract

Stretchable electronic and optoelectronic devices based on controllable ordered buckling structures exhibit superior mechanical stability by retaining their buckling profile without distortion in repeated stretch-release cycles. However, a simple and universal technology to introduce ordered buckling structures into stretchable devices remains a real challenge. Here, a simple and general stencil-pattern transferring technology was applied to stretchable organic light-emitting devices (SOLEDs) and polymer solar cells (SPSCs) to realize an ordered buckling profile. To the best of our knowledge, both the SOLEDs and SPSCs with periodic buckles exhibited the highest mechanical robustness by operating with small performance variations after 20,000 and 12,000 stretch-release cycles between 0% and 20% tensile strain, respectively. Notably, in this work, periodic-buckled structures were introduced into SPSCs for the first time, with the number of stretch-release cycles for the SPSCs improved by two orders of magnitude compared to that for previously reported random-buckled stretchable organic solar cells. The simple method used in this work provides a universal solution for low-cost and high-performance stretchable electronic and optoelectronic devices and promotes the commercial development of stretchable devices in wearable electronics.

## Fabrication: Buckle up for stretchable optoelectronics

A straightforward technique for producing rumpled ultrathin films enables flexible solar cells and light-emitting diodes to survive up to 20 000 stretch-release cycles. One way researchers are improving the strength of devices such as bendable displays is by carving small, strain-accommodating buckle profiles into polymer surfaces using lasers. Jing Feng from Jilin University in Changchun, China, and colleagues have now simplified this fabrication strategy by deploying stencils to deposit periodic metal patterns onto stretched-out elastomeric substrates. When a polymer film device is attached to this surface, it avoids bonding to the metal regions — a situation that causes the thin organic layer to naturally ripple when the tension is released from the substrate. Experiments revealed that the micron-scale buckles enhanced mechanical robustness by two orders of magnitude compared to conventional stretchable solar cells.

## Introduction

Stretchable electronic and optoelectronic devices such as stretchable light-emitting devices^1–7^, solar cells^8–11^, supercapacitors^12–15^, batteries^16–18^, conductors^19–23^, and sensors^24–27^ show great potential for next-generation wearable electronics applications. Among various strategies to realize stretchable devices^28–33^, the creation of a buckling profile in ultrathin devices has attracted much attention because of its independence from materials and the structures of these devices, enabling flexibility for device fabrication^34–43^. Different types of buckled ultrathin film-based stretchable electronic and optoelectronic devices have been reported. The process for forming the buckles determines the morphology for ultrathin films and is important for the performance of stretchable devices. Random buckles can be formed by attaching an ultrathin film onto a pre-strained adhesive and elastomeric substrate, followed by the release of the substrate without any control^34,36,39,40,42^. Small-bending radii in sharp corners and extreme-bending regions result in a large-bending strain, which can damage devices. Additionally, the profile of random buckles might change after cyclic stretching due to the changing random bonding regions between ultrathin devices and the elastomeric substrate. Poor mechanical stability is a fatal disadvantage for random buckling stretchable devices due to the above factors. In contrast, devices based on controllable ordered buckles prepared via sophisticated configuration design and processing can avoid the formation of sharp corners and extreme bending and maintain the ordered buckling profile without distortion, which can much improve their mechanical stability. Introducing well-designed ordered structures on the surface of an adhesive and elastomeric substrate via the use of a programmable laser ablation process has been demonstrated as a feasible strategy to fabricate periodic buckles for stretchable organic light-emitting devices (SOLEDs) with high mechanical stability^41^. However, considering that the laser ablation system and process is both expensive and complicated, a simple and low-cost technology for realizing stretchable devices with ordered buckles remains a real challenge, which is a major obstacle for the practical applications of stretchable devices.

The stencil-pattern transferring technique is commonly used for copying and transferring patterns to target substrates^44–50^. The dimension and size of the fabricated microstructures are the same as the patterns on the stencil. Deposited films with defined patterns can be directly utilized as functional layers, such as patterned metal electrodes in organic optoelectronic devices^51–56^. Here, we developed a simple and general stencil-pattern transferring technique for fabricating ordered buckles in stretchable organic optoelectronic devices. A periodic metal film was deposited onto the surface of an adhesive and elastomeric substrate via a stencil and used as a barrier layer to modify the surface viscidity distribution of the substrate without affecting its elasticity. When an ultrathin optoelectronic device was attached onto the pre-stretched substrate, bonding and nonbonding regions between the device and substrate were defined by the periodic barrier layer. Controllable and ordered buckles have been achieved after releasing the pre-strained substrate. SOLEDs and polymer solar cells (SPSCs) were fabricated by a simple process. Notably, the SPSCs in this work are the first reported SPSCs with periodic-buckled structures. The SOLEDs and SPSCs exhibited outstanding mechanical stability with small performance variations after 20,000 and 12,000 stretch-release cycles between 0% and 20% tensile strain, respectively. The periodic-buckled SPSCs exhibited enhancements of two orders of magnitude in the number of stretch-release cycles compared to that for previously reported random-buckled SPSCs^11,34,43^. The simple and low-cost stencil-pattern transferring technique employed in this work exhibits great potential as a universal solution for various stretchable electronic and optoelectronic devices because of its compatibility with various materials, structures, and fabrication processes for device fabrication; this compatibility is important for promoting the commercial development of stretchable electronic and optoelectronic devices in wearable electronics.

## Materials and methods

### Materials

Polymer substrates for ultrathin OLEDs and PSCs were fabricated by a NOA 63 photoresist, which was purchased from Norland Products Inc. (USA). Adhesive and elastomeric substrates (3 M VHB 4905 tape) and plastic tapes (Scotch Magic Tape) were purchased from the 3 M Company (USA). Metal stencils were custom-made by ZLDSK Corporation (China). MoO_3_, NPB (*N*,*N*′diphenyl-*N*,*N*′-bis(1,1′-biphenyl)-4,4′-diamine), CBP (4,4′-bis(*N*-carbazolyl)-1, 1′-biphenyl), Ir(BT)_2_(acac) (2,3,5,6-tetrakis(3,6-diphenylcarbazol-9-yl)-1,4-dicyanobenzene), TPBi (1,3,5-tris(*N*-phenyl-ben-zimidazol-2-yl)benzene), PCDTBT (poly(*N*-9′-heptadecanyl-2,7-carbazole-alt-5,5-(4′,7-di-2-thienyl-2′,1′,3′-benzothiadiazole))) and PC_71_BM ((6,6)-phenyl C71 butyric acid methyl ester) were purchased from Luminescence Technology Corporation (Taiwan, China). Ca was purchased from Sigma-Aldrich (USA). Ag was purchased from ZhongNuo Advanced Material (Beijing, China) Technology Co., Ltd. All materials for the fabrication of the OLEDs and PSCs were used as received without any treatment.

### Barrier layer fabrication

**Fig. 1:**
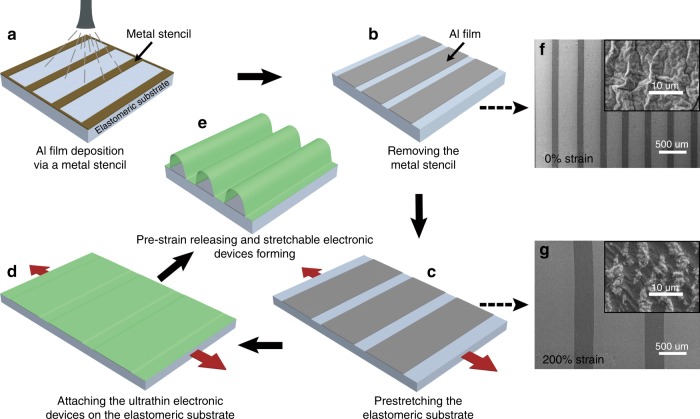
Schematic diagram showing the fabrication process for the stretchable electronic devices. **a** Depositing Al film on an elastomeric substrate by thermal evaporation via a metal stencil. **b** Removing the metal stencil from the elastomeric substrate. **c** Pre-stretching the elastomeric substrate. **d** Attaching an ultrathin electronic device onto the elastomeric substrate. **e** Releasing the pre-strained substrate to form stretchable electronic devices. **f**, **g** SEM images of the periodic Al film deposited onto the elastomeric substrate before and after stretching corresponding to **b** and **c**, respectively. Insets show enlarged SEM images for the periodic Al film deposited onto the elastomeric substrate before and after stretching

The adhesive and elastomeric substrate was cut to the desired size (5 cm × 5 cm), and a working region (0.6 cm × 5 cm) was defined by plastic tape. A metal stencil was attached onto the working region with the long axes parallel to each other. A layer of aluminum (Al) film was deposited onto the surface of the elastomeric substrate via the metal stencil by thermal evaporation (Fig. 1a). The thickness of the Al barrier layer was 50 nm. Then, the metal stencil was removed from the substrate and a periodic Al film was obtained as a barrier layer (Fig. 1b). The regions of the adhesive and elastomeric substrate covered by the Al film lost viscidity. As shown in Fig. 1c, the substrate was pre-stretched, with the period of the Al barrier layer increased.

### SOLEDs fabrication

Ultrathin OLEDs were fabricated by a stripping process^40^. An Ir(BT)_2_(acac)-doped CBP film was used as the emitting layer. The detailed device structure was Ag/MoO_3_/NPB/CBP:Ir(BT)_2_(acac)/TPBi/Ca/Ag, as shown in Supplementary Fig. S1. The free-standing ultrathin OLED was placed onto the surface of a roller and then transferred to the pre-stretched elastomeric substrate (Fig. 1d). During this process, bonding was limited to only the bare regions of the substrate, with no bonding occurring at the Al barrier layer regions. Finally, in Fig. 1e, SOLEDs with periodic buckles were formed after releasing the pre-stretched elastomeric substrate. The original length of the elastomeric substrate was 6 mm, which was increased to 18 mm after stretching to 200% strain. During the buckle forming process shown in Fig. 1d, e, the length of the elastomeric substrate contracted to 9 mm. When re-stretching the devices, the largest tensile strain was 100% according to the length increase from 9 to 18 mm.

For the fabrication process of the SOLED array, the stencil used for Al barrier layer deposition contained a period of 550 μm, of which the width of the open spaces and metal lines were 450 and 100 μm, respectively, as shown in Supplementary Fig. S2. OLED stripes were precisely attached onto the adhesive bare regions of the adhesive to ensure overlap for the period of the OLED array and the Al barrier layer.

### Fabrication of SPSCs

Ultrathin PSCs were fabricated by the same stripping process as used for SOLEDs. NOA 63 film coated onto a silicon slice was first transferred into a vacuum chamber to thermally deposit an Ag cathode and TPBi layer. Then, a solution of PCDTBT:PC_71_BM (1:4 w/w, polymer concentration of 25 mg mL^−1^ in 1,2-dichlorobenzene) was spin-coated atop the TPBi layer. The spin-coating speed was 3000 r.p.m. for half a minute. The resulting film had a thickness of ~80 nm. The whole semi-finished device was annealed on a heating stage at 70 °C for 60 min in a glove box filled with inert gas. Finally, the sample was transferred into the vacuum chamber again to deposit the MoO_3_, Au, and Ag layers. The ultrathin polymer solar cell was peeled off from the silicon slice and attached onto the pre-stretched elastomeric substrate. SPSCs with periodic buckles were formed after releasing the substrate.

### Characterization

All SEM images were taken by a JEOL JSM-7500F scanning electron microscope (SEM) (JEOL Ltd.). An XP-2 stylus profilometer (Ambios Technology, Inc.) was utilized to measure the thickness of the ultrathin NOA63 film. I−L−V characteristics for all OLEDs were investigated by a Keithley 2400 source meter and a Photoresearch PR-655 spectrophotometer with an MS-75 and SL-1× composite lens. PSCs and SPSCs were characterized by a Keithley 2400 source meter under illumination provided by a solar simulator (AM 1.5 Global spectrum, with a light intensity of 100 mW cm^−2^).

## Results and discussion

The metal stencil is shown in Supplementary Fig. S3. The period of the patterns is 400 μm, of which the widths of the open space and the metal line are 300 and 100 μm, respectively. As shown in Fig. 1a, the stencil seamlessly contacted with the adhesive and elastomeric substrate. As a result, the obtained Al barrier layer was uniform and showed the same period as the stencil pattern as shown in the SEM image in Fig. 1f. After stretching the substrate to 200% strain, the period of the Al barrier layer changed (Fig. 1g). In particular, its period increased to 1200 μm, of which the width of the metal stripe region and the bare region were ~900 and 300 μm, respectively. The increasing proportion was identical to the tensile strain, which demonstrated that the deposited thin Al film did not affect the stretchability of the elastomeric substrate. The uniform deformation of the elastomeric substrate was beneficial for ordered buckle formation. The inset of Fig. 1f shows the surface morphology of the thermally evaporated Al film for a zoomed-in region. A large roughness and masses of small random wrinkles can be observed. The morphology of the metal films deposited by thermal evaporation is related to the receiving substrate materials^57–59^. The Al film fractured to small fragments after being stretched to 200% strain (inset of Fig. 1g). The fracture phenomenon was also observed in Al films with different thicknesses, as shown in Supplementary Fig. S4. The width of the Al fragments and their gaps were all smaller than 10 μm with various thicknesses.

**Fig. 2:**
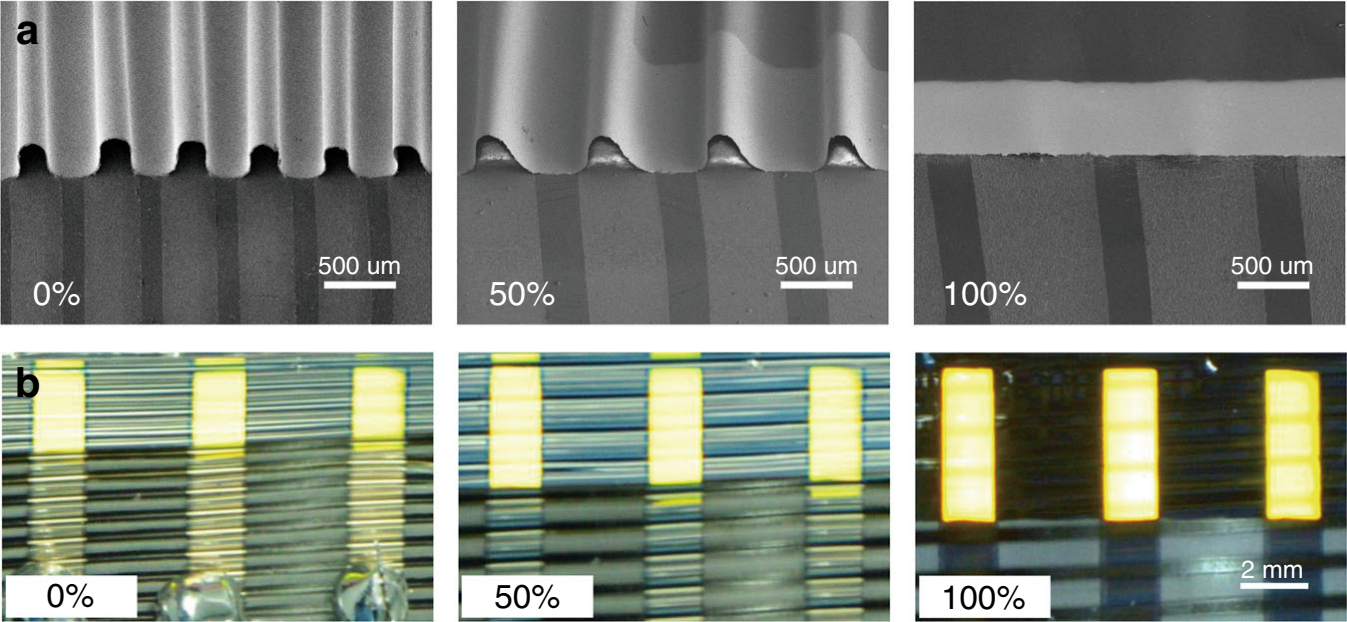
Demonstration of SOLEDs. SEM images (**a**) and photographs (**b**) of the SOLEDs at 0%, 50%, and 100% tensile strain, respectively

SOLEDs and SPSCs based on the stencil-pattern transferring process were fabricated. Ultrathin OLEDs and PSCs were obtained by fabricating devices on ultrathin polymer films. The total thickness of the ultrathin devices was ~3 μm, of which the polymer film thickness was ~2.8 μm. Figure 2 shows the SOLEDs at different tensile strain. The ultrathin OLEDs only adhered to the periodic bare regions and bent above the Al regions of the substrate, as shown in Fig. 2a. Periodic buckles within the whole ultrathin device were formed, which demonstrated effective control of the buckling process through the Al barrier layer. With 200% pre-strain, the SOLEDs exhibited a large tensile strain of 100%. The decreased stretchability was due to incomplete contracting of the elastomeric substrate. The compressive stress of the elastomeric substrate decreased gradually after releasing the pre-strain. At the same time, the small-bending radii resulted in a large-bending stress, with corresponding large resistance in the compressive direction, which hindered the elastomeric substrate from absolutely contracting to its original state. The period of the buckles was the same as that of the Al films at various stretched states. At the largest applied tensile strain of 100%, the periodic buckles disappeared.

**Fig. 3:**
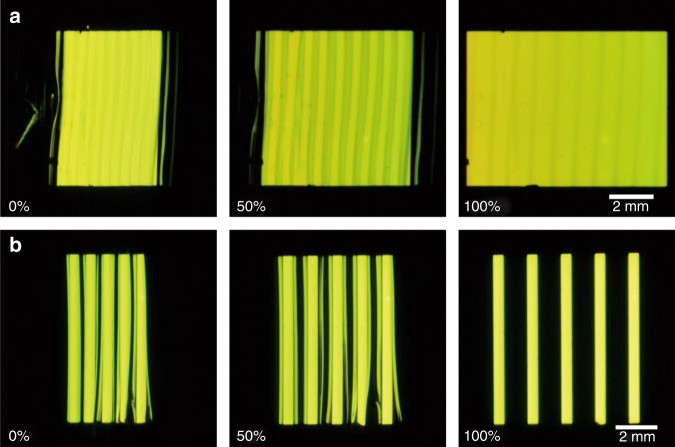
Photographs of the SOLEDs in a dark environment. **a** A large-area SOLED at 0%, 50%, and 100% tensile stain. **b** An array of five OLED pixels at 0%, 50%, and 100% tensile strain

Figure 2b and Supplementary Movie 1 show the operating SOLEDs in static and dynamic states, respectively. Ir(BT)_2_(acac) was used as the light-emitting material, with yellow emission realized. The bright light-emission area gradually elongated with increasing tensile strain, which visually showed the stretchability of the SOLEDs. Periodic buckles and the Al barrier layer can be clearly seen. The operation of the SOLEDs in a dark environment was also investigated, as shown in Fig. 3. Figure 3a shows a large-area SOLED containing nine periods of buckles at different strains. The visible periodic buckles slightly influence the uniformity of the emission observed from the SOLED. Figure 3b (Supplementary Movie 2) shows an array of five OLED pixels; this array forms a pattern of light and dark stripes. The period of the array was the same as that of the buckles. Periodic buckles formed at lower strain and light from each pixel was reflected by an adjacent non-planar metal anode film, which made each pixel look wider. The synchronization of the periodic buckles and the OLED array reveal the potential applications for the SOLED in stretchable displays.

**Fig. 4:**
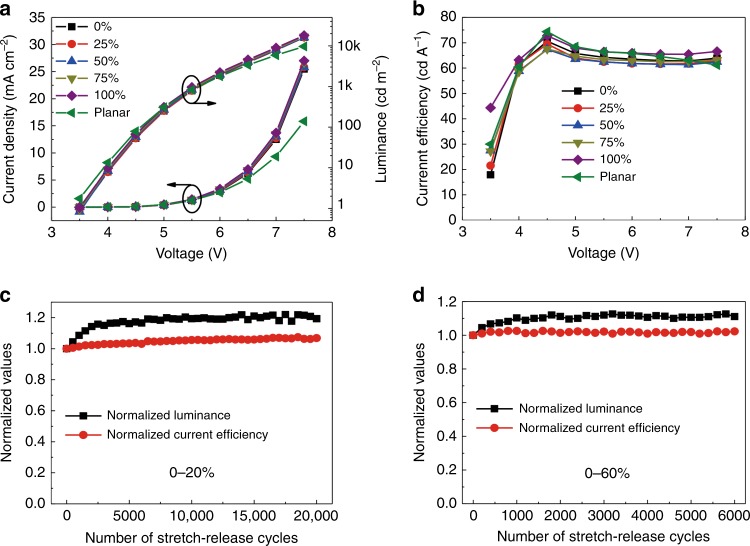
EL performance of SOLEDs. Current density-luminance-voltage characteristic curves (**a**) and current efficiency-voltage characteristic curves (**b**) for the SOLEDs. Device performance under a cyclic stretching test between 0 and 20% strain (**c**) and between 0 and 60% strain (**d**)

The electroluminescence (EL) performance of the SOLEDs was measured at different stretched states, as shown in Fig. 4a, b. The EL performance of the SOLEDs was excellent, stable at different tensile strain and comparable to that of the planar device. The maximum current efficiency at 0% and 100% tensile strain was 71 and 73 cd A^−1^, respectively, with a variation of only 2.8%. Supplementary Figure S5 shows the EL spectra measured at different strain values. A redshift in the spectral curves was observed with increasing tensile strain, which results from the microcavity effect in SOLEDs^41^. The slight redshift (~5 nm) was in agreement with the stable current efficiency. The above results indicate that the periodic buckling profile introduced into the SOLEDs leads to negligible deterioration of the device performance.

The mechanical stability of the SOLEDs was examined by cyclically stretching the devices between varying strain values. The SOLED was measured at 0% strain after every 500 stretch-release cycles in air without any encapsulation. The driving voltage was 5.5 V. Figure 4c, d shows the EL performance of the SOLEDs. The luminance increased by ~20% during the first 3000 cycles of cyclic stretching and became stable in the following test between 0 and 20% strain (Fig. 4c). Similarly, the luminance of SOLEDs slightly increased during cyclic stretching between 0 and 60% strain, as shown in Fig. 4d. The current efficiency exhibited a slight variation of 2%. As seen from Supplementary Figs. S6 and S7, the normalized current density for the SOLEDs under the cyclic stretching test showed a similar increase during the initial 3000 cycles. A possible origin for the increased current density for the initial 3000 stretch-release cycles is the compression effect in small-molecule semiconductor films, as shown in Supplementary Fig. S8^60,61^. The SOLED was found to be mechanically robust, as demonstrated by the cyclic stretching tests. The mechanical robustness was attributed to the ordered buckles. The Al films degraded little and maintained good adhesion with the elastomeric substrate after 20,000 cycles of cyclic stretching (Supplementary Fig. S9). The SOLEDs retained an ordered buckling profile without distortion during the stretch-release process (Supplementary Fig. S10). Any defects in the device can be spread and amplified after thousands of stretch-release cycles, resulting in degraded performance. The smallest bending radius for the periodic buckles was ~100 μm at 0% strain, as shown in Fig. 2a. The bending strain determined by the bending radius and thickness of the ultrathin OLEDs was only ~0.68% for the metallic anode^40^. Therefore, the bending deformation due to the periodic buckles did not cause obvious mechanical and electrical damage to the SOLEDs, and they could operate stably and efficiently with such large-bending strain during cyclic stretching.

**Fig. 5:**
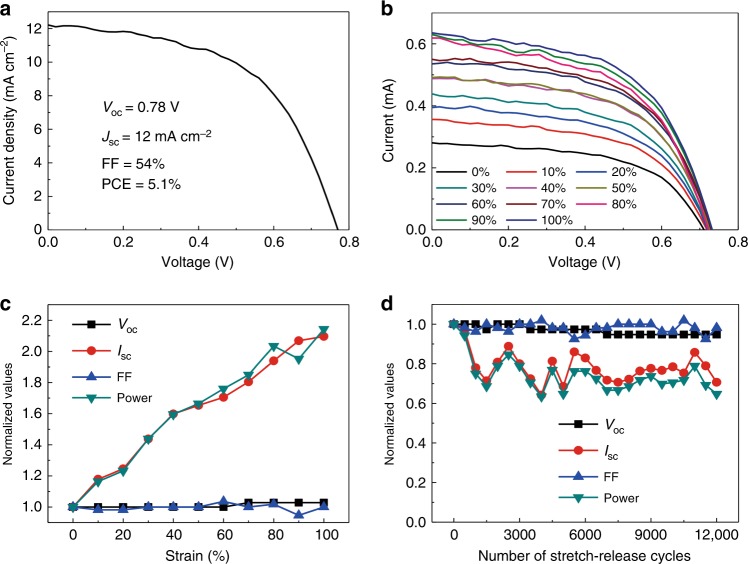
Characterization of SPSCs. **a** *J*–*V* characteristic curve for a planar polymer solar cell. **b** *J*–*V* characteristic curves for the SPSCs at different strain values. **c** Parameters for the SPSCs at different strain values. **d** Device performance under a cyclic stretching test between 0 and 20% strain

Stretchable solar cells are an important member of stretchable organic optoelectronics. Efficient and mechanically robust stretchable solar cells are the best candidates for electric power suppliers in a stretchable electronic system integrated with other stretchable electronic devices. However, previously reported stretchable solar cells with random buckles could only withstand tens to hundreds of cyclic stretches, which hindered their use for commercial applications^11,34,39,43^. Here, we demonstrated mechanically robust SPSCs based on the use of a simple stencil-pattern transferring technology. This paper presents the first report of SPSCs with periodic buckling structures. The device structure of an ultrathin polymer solar cell is shown in Supplementary Fig. S11. A polymer-fullerene derivative PCDTBT:PC_71_BM blend was used as light absorber. The SPSCs were also fabricated by following the steps shown in Fig. 1a–e. Periodic buckles were formed, with a large tensile strain of up to 100%, as shown in Supplementary Fig. S12. The *J*–*V* characteristic curve for a planar solar cell is shown in Fig. 5a. Its power conversion efficiency was 5.1%, with an open-circuit voltage (*V*_OC_) of 0.78 V, short-circuit current density (*J*_SC_) of 12 mA cm^−2^, and fill factors (FFs) of 54%, which are comparable with the best reported polymer solar cells based on PCDTBT:PC_71_BM^62,63^. The *I*–*V* characteristics for the SPSCs were measured for varying tensile strain values, as shown in Fig. 5b. The devices worked functionally at each stretched state. The normalized values for *V*_OC_, *I*_SC_, FF, and output power at different tensile strain are summarized in Fig. 5c. The output power was defined as

1 $${\mathrm{Output}}\,{\mathrm{power}} = {{V}}_{{\mathrm{OC}}} \times {{I}}_{{\mathrm{SC}}} \times {\mathrm{FF}}$$

*V*_OC_ and FF changed little, while *I*_SC_ and the output power increased almost linearly with increasing tensile strain. This outcome was attributed to the increased light incident area, with tensile strain increasing despite the practical device area not changing. At the same time, the linearly increased *I*_SC_ indicated that the periodic buckles had nearly no influence on the light absorption in the SPSCs.

The mechanical stability of the SPSCs was examined by cyclically stretching the devices between 0 and 20% tensile strain, as shown in Fig. 5d. The *I*–*V* characteristics were measured at 0% strain after every 500 stretch-release cycles. The SPSCs could survive 12,000 stretch-release cycles; this value is two orders of magnitude higher than that reported in previous results obtained for random-buckle-based SPSCs^11,34,43^. To the best of our knowledge, our mechanical stability for SPSCs is the highest reported to date. It should be noted that the degradation in *V*_OC_ and FF was quite small. The stable FF indicated that no large damage due to cracks or delamination was introduced into the PSCs during the cycling, so as not to affect the conductivity and series resistance. The clearly fluctuant *I*_SC_ might be caused by the fluctuant incident area for the SPSCs at 0% strain under each measurement. The elastomeric substrate is visco-elastic. Thus, it would take some time to contract to 0% strain state absolutely, especially under continuously cyclic stretching. Therefore, a small variation exists in the incident area for each measurement during the cyclic stretching test, leading to the observed fluctuation in *I*_SC_.

## Conclusions

A simple and universal stencil-pattern transferring technology was applied to stretchable organic optoelectronic devices, resulting in the demonstration of high-performance SOLEDs and SPSCs with periodic buckles. A periodic Al barrier layer that was deposited onto elastomeric substrates by a stencil pattern played the role of forming periodic buckles for ultrathin optoelectronic devices. The SOLEDs and SPSCs both exhibited the highest mechanical robustness reported to date; notably, the SPSCs showed an improvement of two orders of magnitude. Stretchability, efficiency, mechanical stability, and production cost are key factors for practical applications of stretchable optoelectronics. The simple and universal stencil-pattern transferring technology used in this work provides a solution for low-cost and high-performance stretchable optoelectronic devices and demonstrates potential for commercial development of stretchable electronics and optoelectronics.

## Electronic supplementary material

Supporting Information

Supplementary Movie S1

Supplementary Movie S2
