# Supplementary material for: Mechanically robust stretchable organic optoelectronic devices built using a simple and universal stencil-pattern transferring technology
Source: Light Sci Appl. 2018 Jul 4;7:35. doi: 10.1038/s41377-018-0041-x (PMC6106994; doi:10.1038/s41377-018-0041-x)
Supplement: Supplementary file 1 — Supporting Information [file 41377_2018_41_MOESM1_ESM.doc]

Supporting information for

**Mechanically robust stretchable organic optoelectronic devices built using a simple and universal stencil-pattern transferring technology**

Da Yin^1^, Nai-Rong Jiang^1^, Yue-Feng Liu^1^, Xu-Lin Zhang^1^, Ai-Wu Li^1^, Jing Feng^1^ and Hong-Bo Sun^1,2^

^1^State Key Laboratory of Integrated Optoelectronics, College of Electronic Science and Engineering, Jilin University, 2699 Qianjin Street, Changchun 130012, China

^2^State Key Laboratory of Precision Measurement Technology and Instruments, Department of Precision Instrument, Tsinghua University, Haidian, Beijing 100084, China

*Correspondence:*

*Jing Feng, Email:* [*jingfeng@jlu.edu.cn*](mailto:jingfeng@jlu.edu.cn)*;*


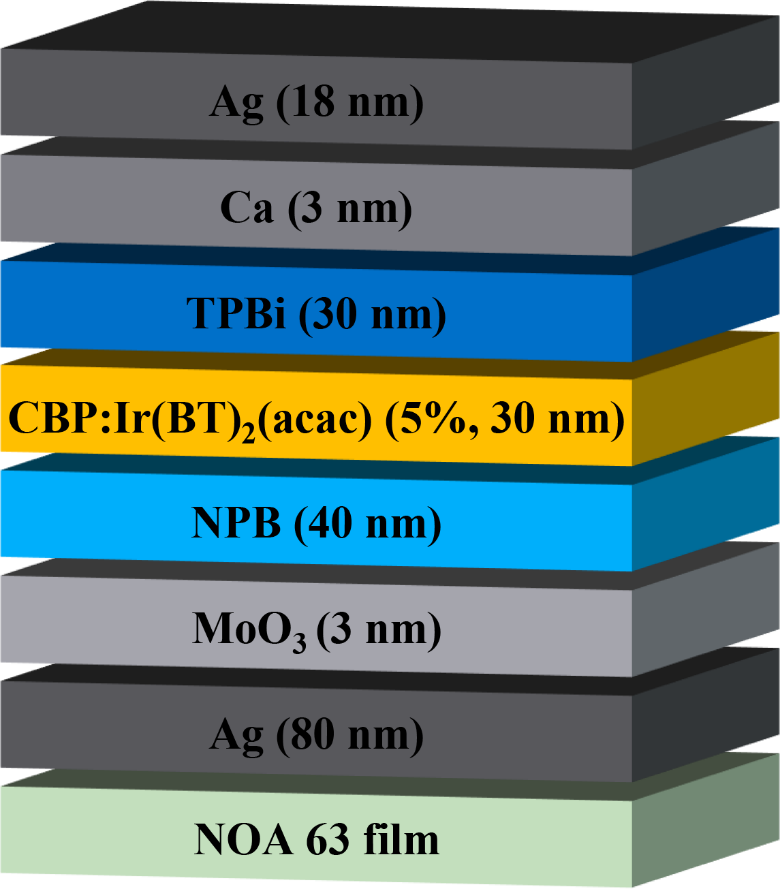


**Supplementary Figure S1** **|** **Device structure of the ultrathin OLED.** The total thickness of the ultrathin device is about 3 μm, in which the NOA 63 film is about 2.8 μm.


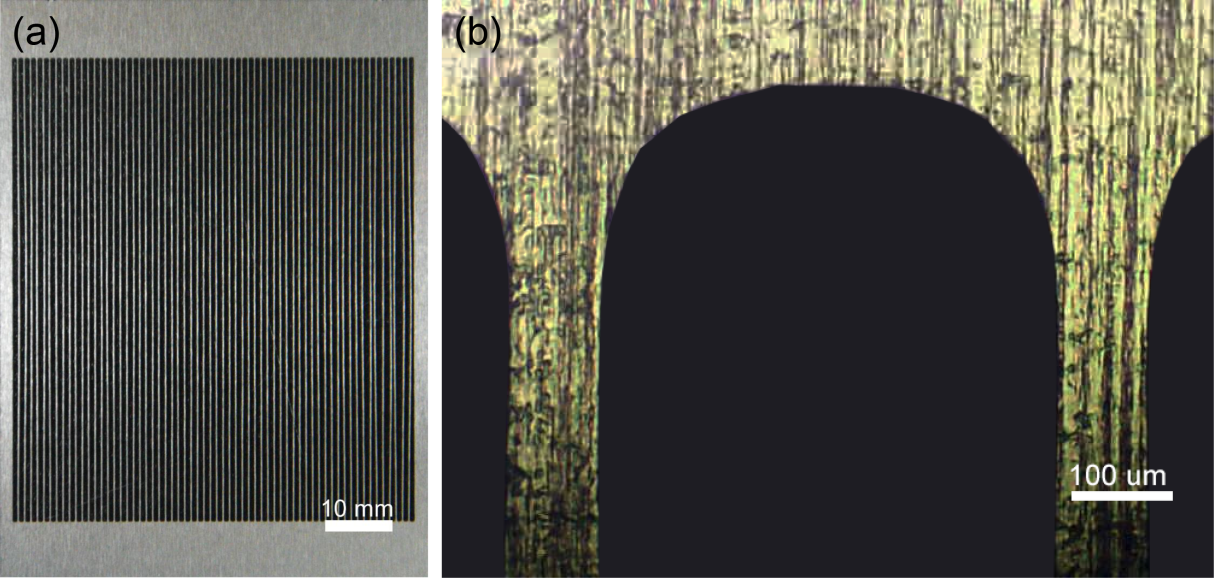


**Supplementary Figure S2 | Metal stencil.** Photograph (a) and optical microscope image (b) of the metal stencil with a period of 550 μm.


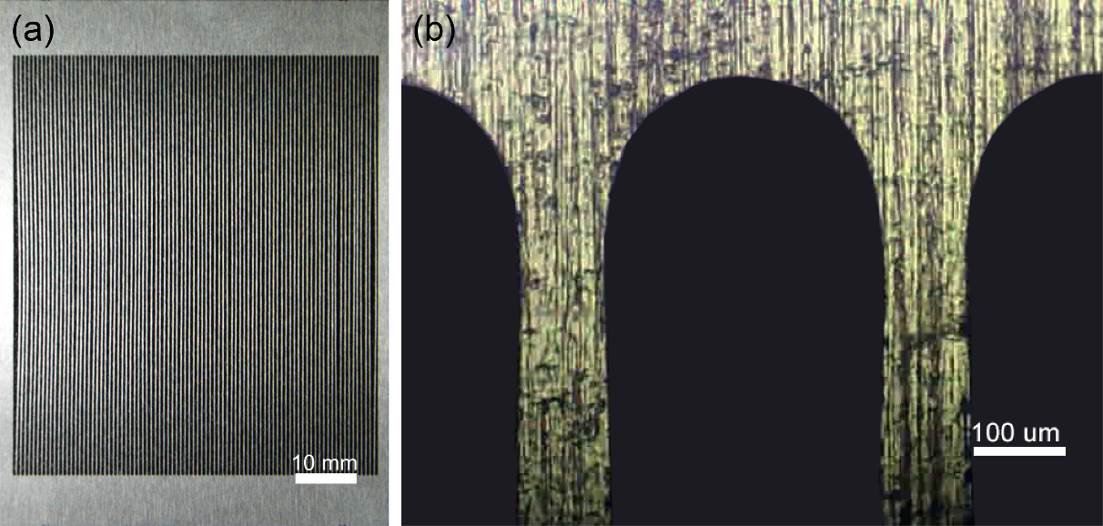


**Supplementary Figure S3 |** **Metal stencil.** Photograph (a) and optical microscope image (b) of the metal stencil with a period of 400 μm.


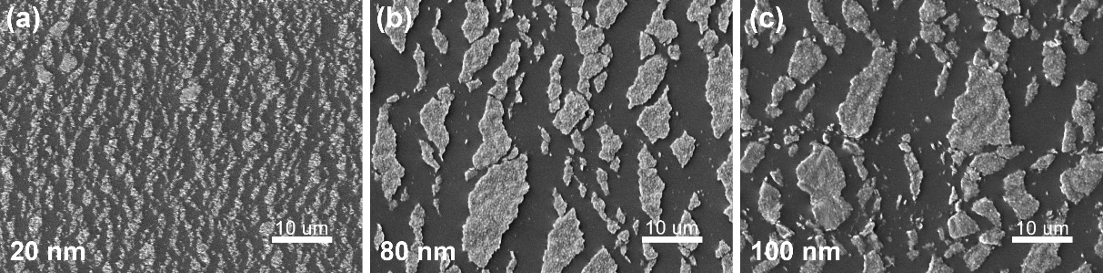


**Supplementary Figure S4 |** **SEM images of fractured Al films.** Al film thermally evaporated on the surface of the elastomeric substrate with thickness of 20 nm (a), 80 nm (b) and 100 nm (c) after stretched to 200% strain.


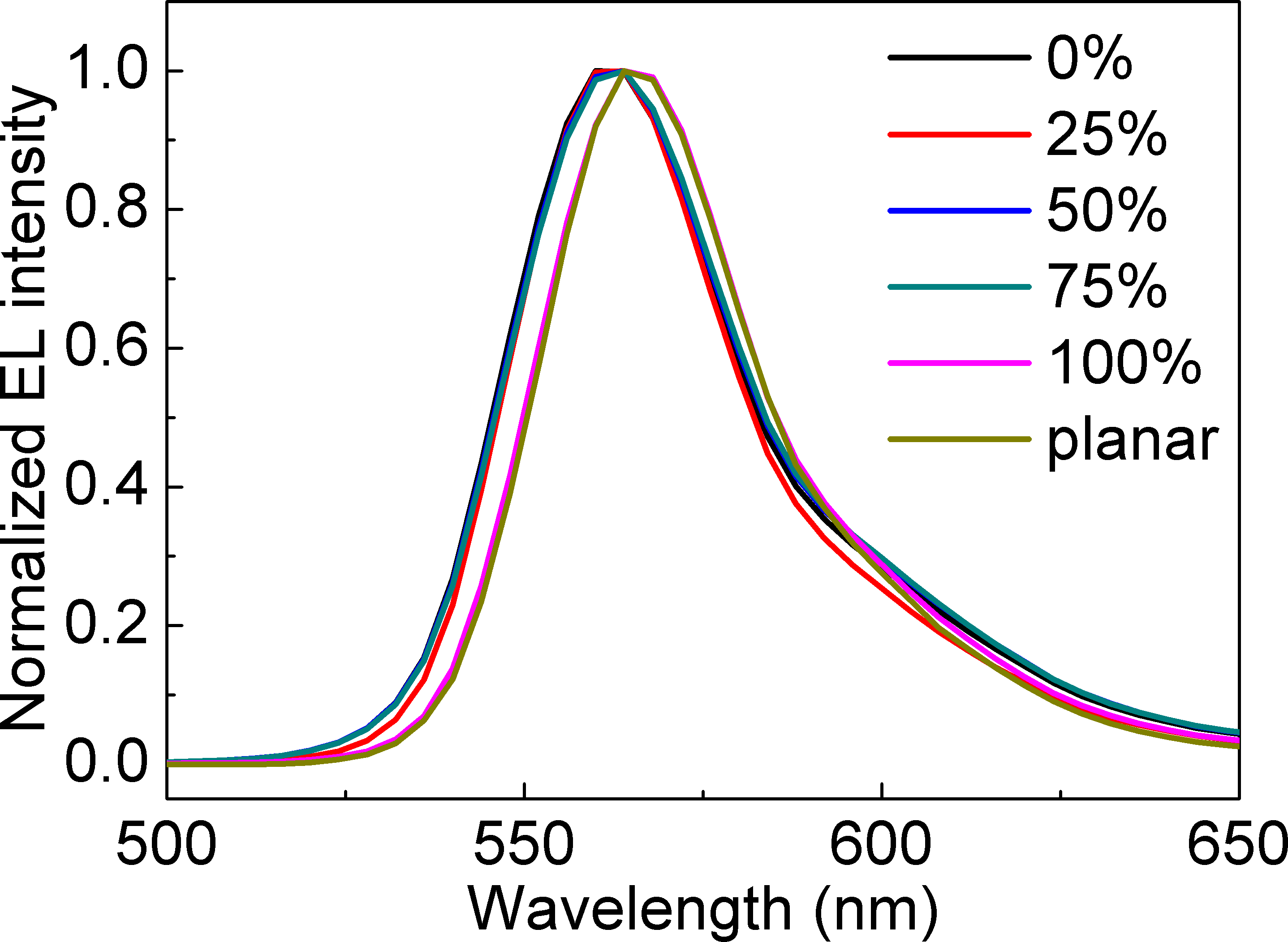


**Supplementary Figure S5 |** **EL spectra of the SOLEDs.** EL spectra of the SOLEDs at 0%, 25%, 50%, 75% and 100% tensile strain. EL spectrum of a planar OLED is plotted for comperation.


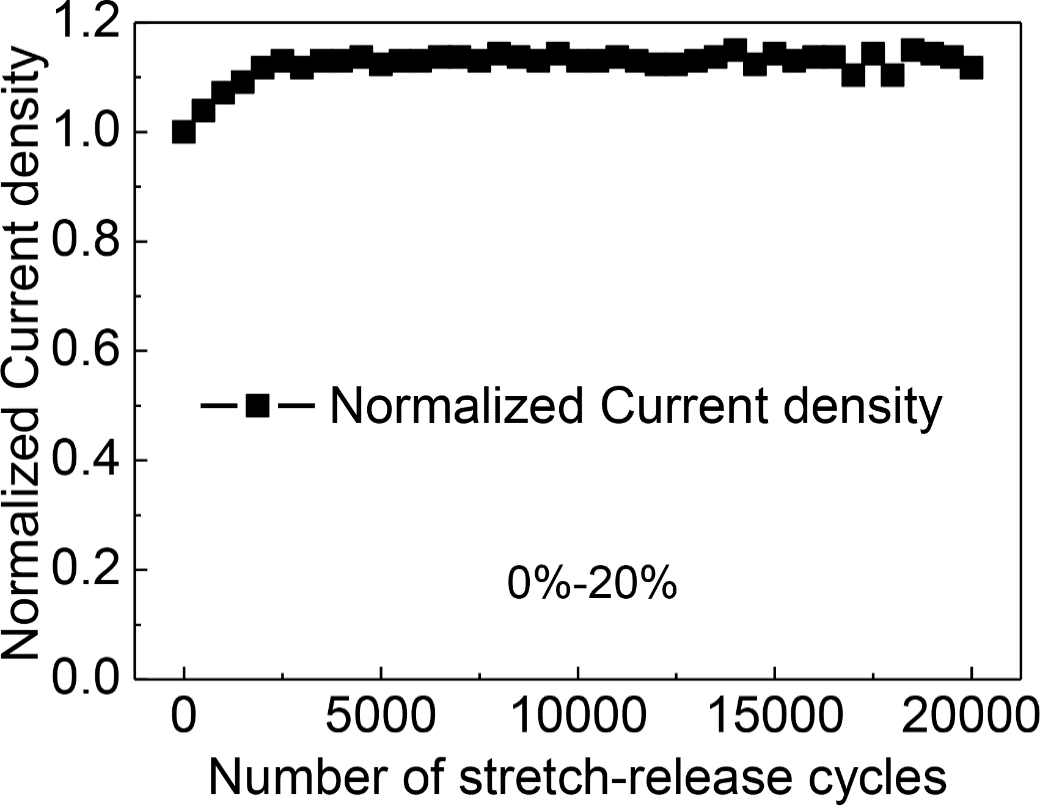


**Supplementary Figure S6 |** **The mechanical stability of the SOLEDs.** Normalized current density of the SOLEDs under cyclic stretching test between 0% and 20% strain.


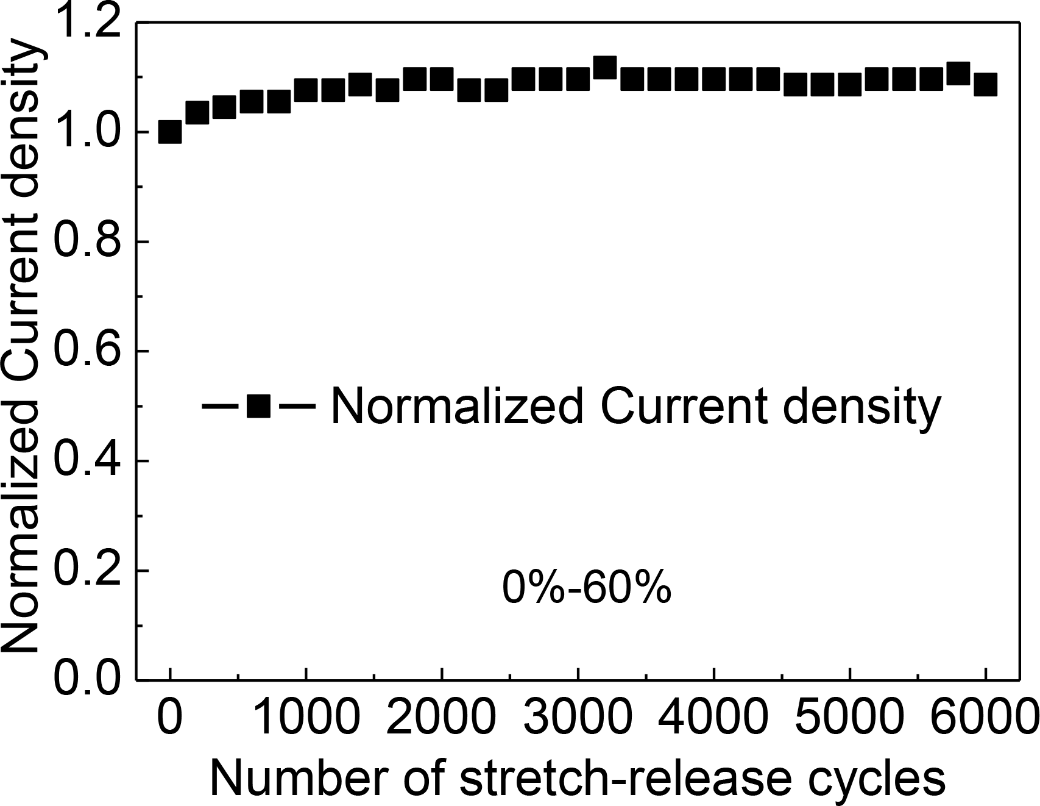


**Supplementary Figure S7 |** **The mechanical stability of the SOLEDs.** Normalized current density of the SOLEDs under cyclic stretching test between 0% and 60% strain.


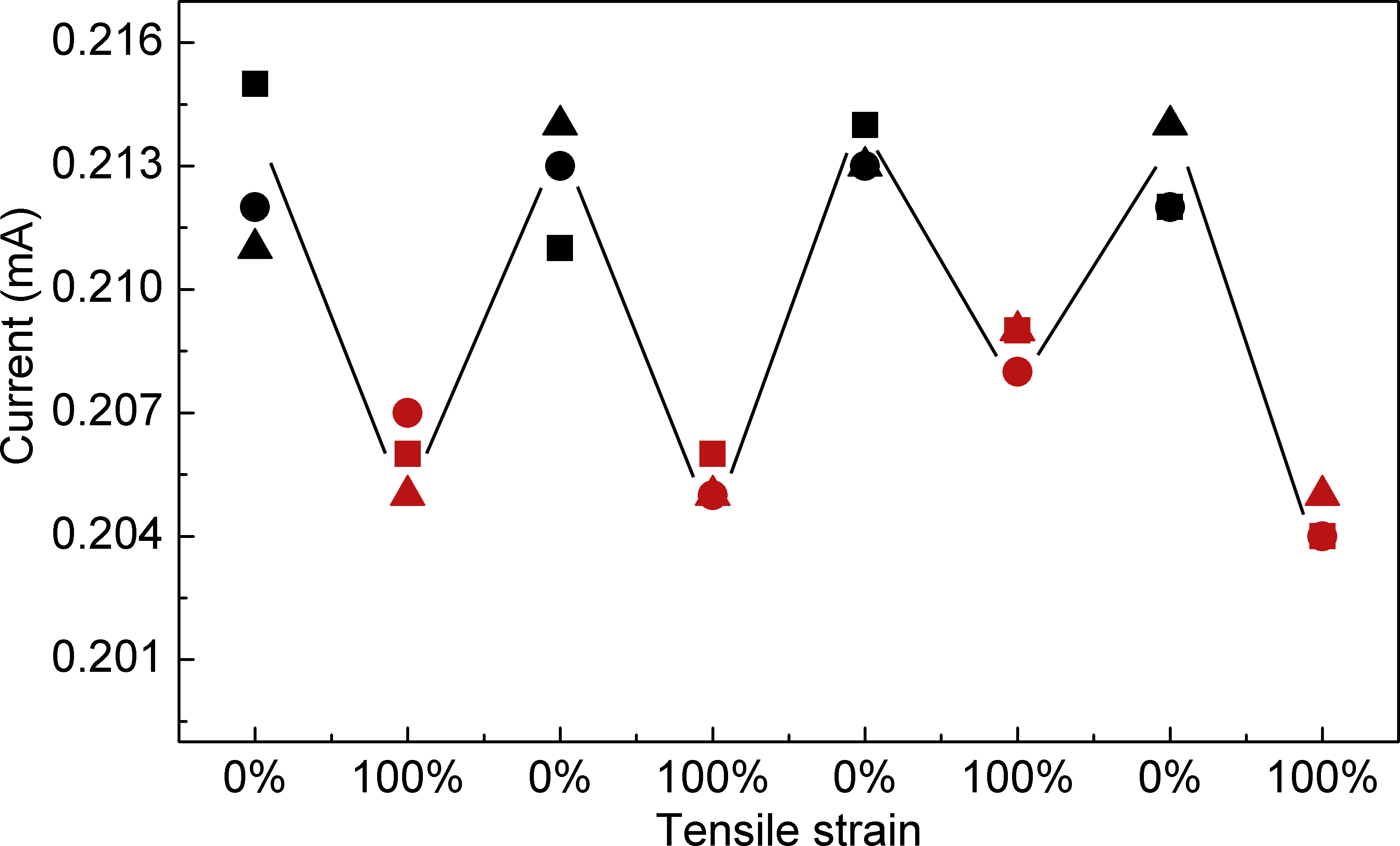


**Supplementary Figure S8 | Compressive effect in a SOLED.** The currents of a SOLED repeatedly measured at 0% and 100% strain respectively. At every strain state, the device was measured at 6 V three times.

Supplementary Figure S6 and S7 are the normalized current density of SOLEDs under cyclic stretching test. These two curves show a similar ascent in the initial stage with the luminance curves. The increasing of luminance is mainly due to the increasing of current density at constant driving voltage during the stretch-release cycles. The layered organic materials of the SOLEDs were compressed in the normal direction at regions with small bending radius, which was the possible origin of the increased current density at the initial 3000 stretch-release cycles. The compression resulted in a denser packing of organic molecules, leading to an increase in conductivity and a better charge transport. The current density increased at a constant driving voltage consequently. Supplementary Figure S8 shows the repeatedly measured current of a SOLED at 6 V at 0% and 100% strain respectively. At these two extreme states, the OLED was compressed under the largest and smallest pressure respectively. It can be seen that the currents at 0% strain were a little larger than those at 100% strain at different measurements, which demonstrated the compression effect. During cyclic stretching, the organic layers of the OLED were compressed gradually and reached the largest compressive state after about 3000 times. As a result, the current density and luminance increased for the first 3000 cycles and then stabilized.


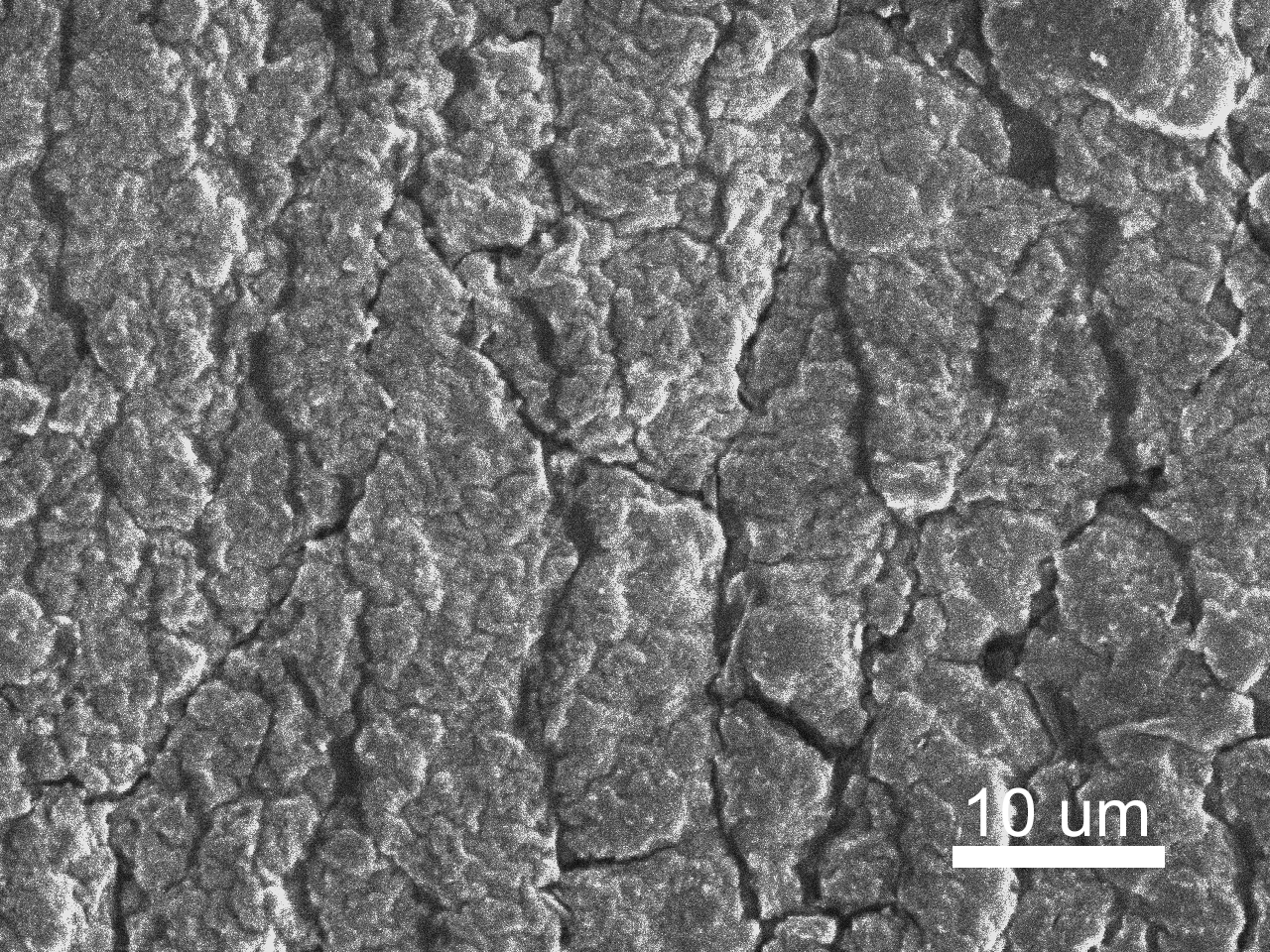


**Supplementary Figure S9 | SEM image of Al film.** SEM image of 50 nm Al film at 0% strain after 20000 times of cyclic stretching between 0% and 20% strain.


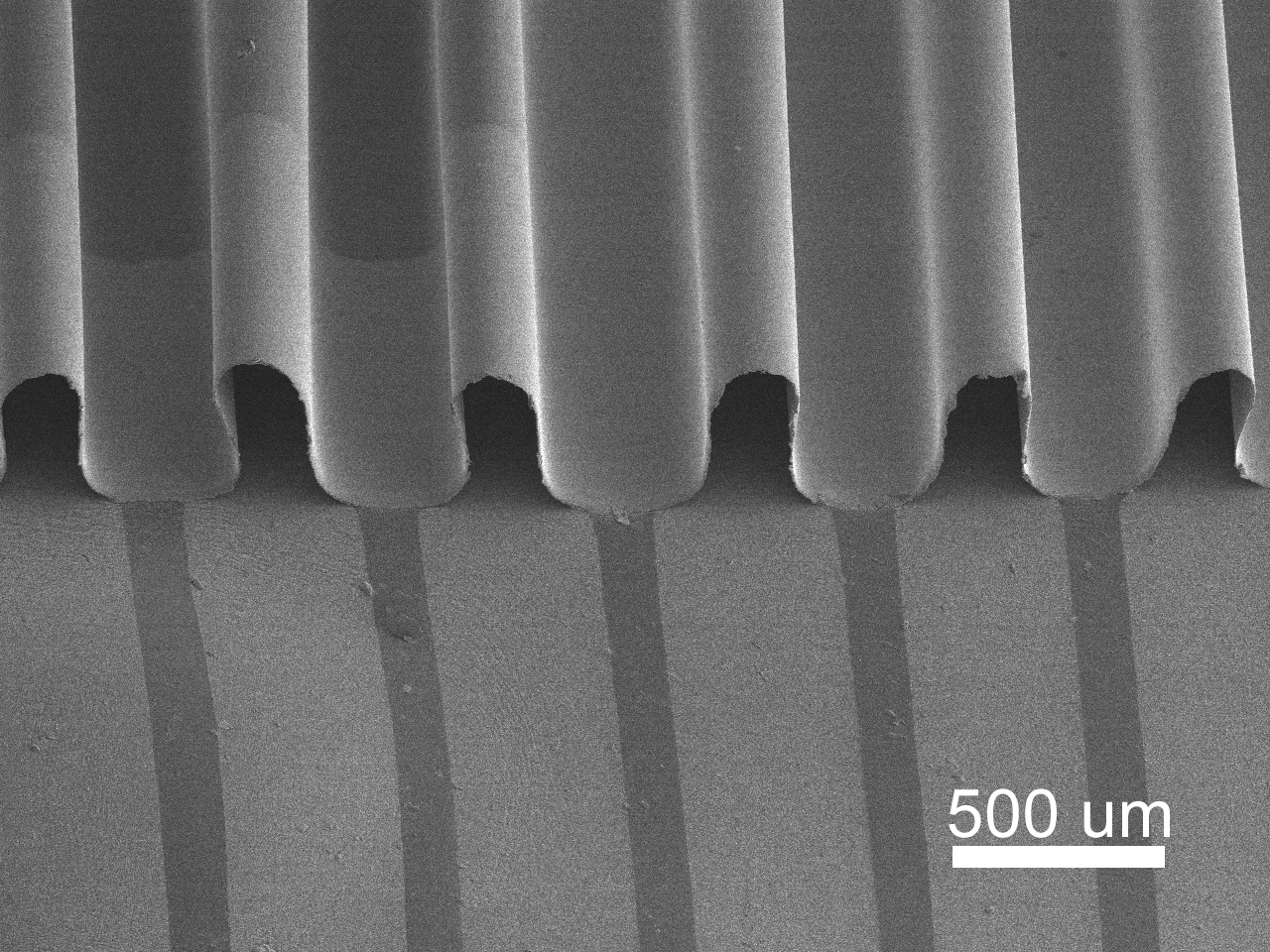


**Supplementary Figure S10 | SEM image of SOLEDs.** SEM image of SOLEDs at 0% strain after 20000 times of cyclic stretching between 0% and 20% strain.


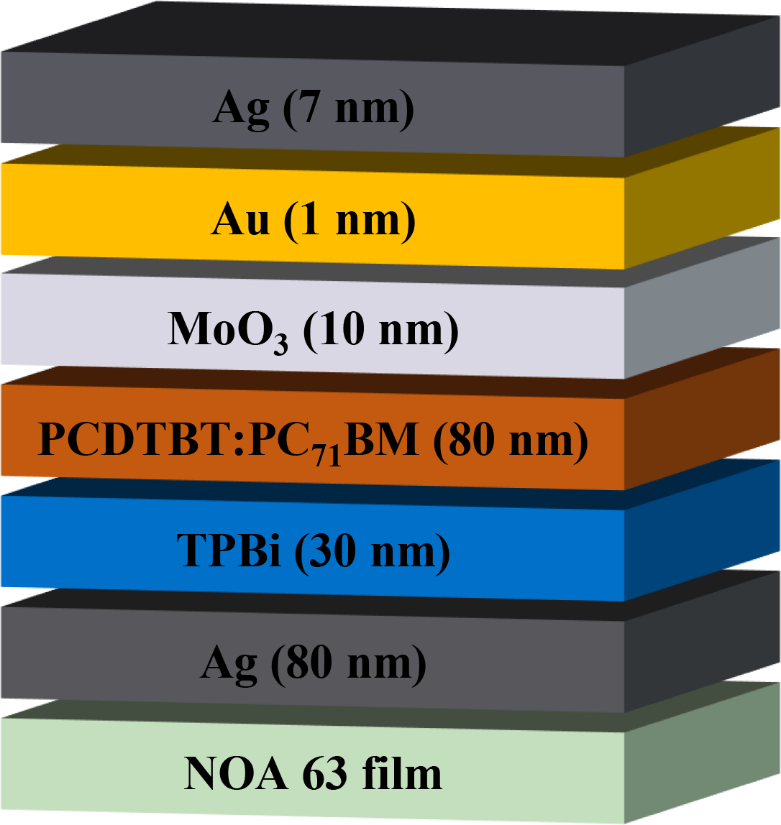


**Supplementary Figure S11 |** **Device structure of the ultrathin polymer solar cell.** An ultrathin Au (1nm)/Ag (7 nm) composite film is used as the anode.


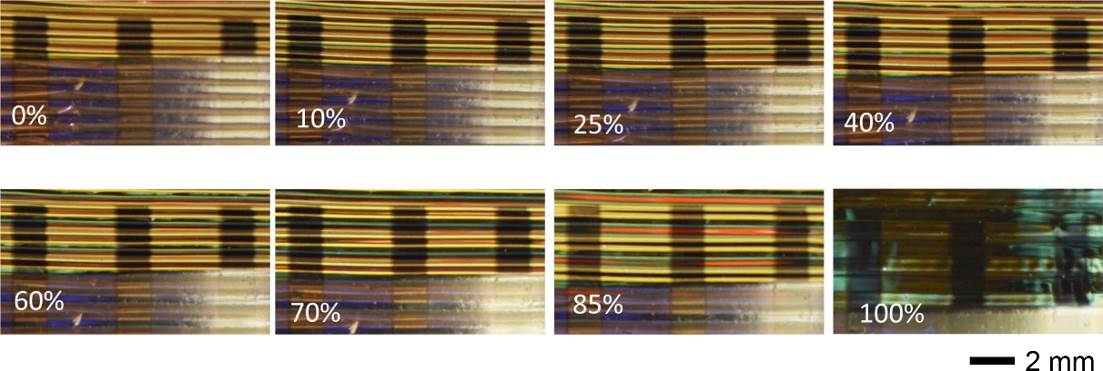


**Supplementary Figure S12 |** **Photographs of SPSCs at different tensile strain.** The SPSCs can be stretched to 100% strain.

**References**

1 Zhong G-Y, Liu Y, Song J, Zhao Q, Li Y-S *et al.* Strong piezoresistance effect of small molecule organic semiconductors. *J Phys D: Appl Phys* 2008; **41**: 205106.

2 Kim JH, Seo SM, Lee HH. Nanovoid nature and compression effects in organic light emitting diode. *Appl Phys Lett* 2007; **90**: 143521.
